# Investigating the Impact of Occupational Technostress and Psychological Restorativeness of Natural Spaces on Work Engagement and Work–Life Balance Satisfaction

**DOI:** 10.3390/ijerph20032249

**Published:** 2023-01-27

**Authors:** Matteo Curcuruto, Sian Williams, Margherita Brondino, Andrea Bazzoli

**Affiliations:** 1 School of Humanities and Social Sciences, Leeds Beckett University, Leeds LS1 3HE, UK; 2 Department of Human Sciences, University of Verona, 37129 Verona, Italy; 3 Department of Psychology, Washington State University, Vancouver, WA 98686, USA

**Keywords:** environmental psychology, remote working, technostress, nature restorativeness, work engagement, work–life balance

## Abstract

The COVID-19 pandemic has necessitated lockdowns and mandatory working from home, as well as restrictions on travel and recreation. As a result, many people have had to use their home as an office and have increased their use of Information Communications Technology (ICT) for work purposes. Nature and accessing natural spaces are known to be beneficial for human health and wellbeing, as a result of their restorative properties. Access to local outdoor spaces was permitted under restrictions, and use of such spaces increased during lockdown. This survey study investigated whether the perceived restorativeness of natural spaces and exposure to technostress predicted the levels of work engagement and work–life balance satisfaction (WLBS) during the period of COVID-19 restrictions adopted in 2020. Analyses conducted on a sample of 109 people employed in the UK revealed that technostress negatively impacted WLBS, whilst perceived psychological restorativeness positively predicted work engagement. The study highlights the benefits of having access to natural spaces to improve employees’ work engagement and potentially negate the negative effects of technostress, particularly during a period of intensive working from home. The results contribute to the understanding of the linkages between restorativeness and work engagement, paving the way for synergies across these research fields.

## 1. Introduction

One of the early responses to the recent SARS-CoV-2 (“COVID-19”) pandemic was a sudden and rapid shift to home working for many office-based roles [1]. Many public and private institutions were obliged to introduce a massive deployment of remote, working-from-home arrangements, mediated by Information and Communications Technology (ICT), which inevitably changed the definition, perception and experience that individuals have regarding their personal work environment [2]. This dramatic change impacting the occupational experience and daily life routines of individuals gives rise to different research questions concerning how these modifications in the work experience have potentially impacted the life of millions of individuals.

In addition to the other academic approaches that contribute to generating research insight on the promotion of improvements in the quality of work life experience [3], the psychology of workplace environment is a rich and diverse field of study that is growing quickly [4]. The knowledge developed in this field concerns the effects of the workplace experience and its environmental features on worker morale and productivity [5,6]. The first main research aim of the present study is the investigation of how work engagement and work–life balance satisfaction were affected by the prolonged lockdown experience of ‘remote work from home’ arrangements mediated by ICT tools, such as Microsoft Teams, Skype, mobile hardware and other technological systems, which were deployed by organizations to ensure the continuity of their business during the national lockdown period. Recent studies have already evidenced how prolonged working-from-home arrangements might contribute to an increase in the risk of technostress [7]. Technostress is a form of occupational stress that is associated with ICT, such as the internet, mobile devices and other social media. Even in normal times, technostress can be seen in many organizations at all levels, with affected employees becoming anxious or overwhelmed by working in computer-mediated environments in which there is a constant flow of new information. We can assume that in a time characterized by a massive usage of ICT technologies to mediate working-from-home arrangements, technostress may have a detrimental effect on individual work engagement and work–life balance satisfaction.

On the other side, the pandemic also resulted in increased use of local natural spaces for exercise and recreation [8], especially sites which can be accessed by foot or bike [9], due to the closure of indoor facilities and travel restrictions. Increasingly, time in nature is being advocated as a method to mitigate the impacts of stress, allowing for a break from virtual environments and reconnection with natural spaces and their therapeutic effects [10]. Therefore, in parallel to the investigation of the impact of technostress on individual work engagement and work–life balance satisfaction, the second main research aim of this study is to explore if access to natural environments during the prolonged lockdown period positively influenced the same psychological dimensions of engagement and work–life balance satisfaction, thanks to the psychologically restorative properties of natural environments. By definition, restorative environments are environments that facilitate the recovery of biological, psychological, cognitive and social resources in individuals. An increasing number of studies from different areas (environmental psychology, health psychology, organizational psychology) indicate that the exposure to natural environments can influence people’s wellbeing [5]. The second research question of the present project is to assess if access to outdoor natural restorative environments during the third national lockdown in the United Kingdom produced wellbeing benefits to the people who were operating under “remote work arrangements”. In summary, this study aimed to examine whether the perceived restorative effects of nature have benefited employee work engagement and work–life balance satisfaction during a time characterized by a generalized usage of remote working and an intense usage of IT technologies, as a result of the global COVID-19 pandemic.

In the following sections, we will first review the notion of technostress and how it could have negatively impacted employees’ work engagement and work–life balance satisfaction during a prolonged period of remote working. Then, we will review current literature on the psychological restorativeness of natural environments and how access to natural environments during the prolonged time of remote working mediated by ICT technologies could have sustained the same psychological states of work engagement and work–life balance satisfaction, in spite of the potential concurrent effects induced by the experience of technostress.

The Phenomenon of Technostress. Workplace stress is a well-recognised phenomenon, with many factors contributing to employee stress levels [11]. One such stressor is the use of Information Communications Technology (ICT), which enables constant connectivity to online functions. Studies have shown that anxiety, pressure, dissatisfaction and confusion about job demands can all result from intensive ICT use [12]. This phenomenon is referred to as “technostress” [13] and can cause information fatigue, loss of motivation and dissatisfaction at work [14,15]. Tarafdar et al. [12] described five technostressors: overload, invasion, complexity, insecurity and uncertainty. These stressors contribute to physical [16] and mental impacts upon employees [17], which can lead to decreased engagement with work, absenteeism and general dissatisfaction [18]. With the move to home working for many during the pandemic, particular characteristics of technostress, such as intrusiveness and constant connectivity [14], may reasonably be considered to have increased as the line between home and office became blurred [19,20]. In the present study, we aim to understand the influence of technostress on two positive psychological states: work engagement and work–life balance satisfaction. Measuring engagement with work is one way by which the impacts of technostress can be assessed in occupational contexts. This study uses Schaufeli et al.’s [21] definition of work engagement as being a work-related psychological state, which is “positive and fulfilling”, characterised by “vigour, dedication, and absorption”. Engaged employees have high energy levels and are immersed in, and enthusiastic about, their work [22]. Molino et al. [23] highlighted increased technostress levels during the pandemic; therefore, assessing whether technostress has any effect on work engagement and life satisfaction during periods of home working is a key part of this research. Work–life balance satisfaction (WLBS) is a concept that refers to the equilibrium between personal life and career [24]. As technostress is known to impinge on personal life, blur boundaries between home and work and lead to longer-term health issues [14], it is hypothesised that higher levels of technostress will negatively affect participants’ WLBS. In line with these conceptual reflections, we advance our first set of research hypotheses concerning the influence of technostress.

**Hypothesis** **1.** *The subjective experience of technostress will negatively influence work engagement (h1a) and work–life balance satisfaction (h1b).*

Nature, Health and Restorative Environments. There is an extensive and rapidly growing body of literature on the benefits of accessing the natural environment for health and wellbeing [25]. A seminal study in 1984 by Ulrich [26] was among the first to provide empirical evidence that exposure to nature improves human health. More recent studies have demonstrated that time spent in “the natural environment”, including wild places [27], greenspaces [28], blue spaces [29] and “in-between” spaces, such as graveyards [30], can provide improvements in health measures, including clinical and wellbeing indicators, epidemiological measures and public-health statistics [31]. The mechanisms that deliver these outcomes are complex, with research indicating a wide range of potential causal factors, including exposure to calming soundscapes, natural patterns (fractals) and chemicals, which have beneficial effects, increased physical activity and social aspects, such as meeting with friends in outdoor spaces [32]. There is consensus that the outdoor environment generally acts as a restorative environment, i.e., one which enables an individual to psychologically and/or physiologically recover from stressors and fatigue [33]. Such environments lead to improved performance in attention tasks, mood measures and reduced signs of physiological stress (e.g., heart rate, blood pressure, cortisol levels) [34]. Natural spaces frequently score highly for restorative effects and have been shown to be more restorative than urban environments [35]. Research on the mental health benefits of natural environments has been driven by two theories. Stress Recovery Theory (SRT) [36] concerns restoration from stress after a demanding or threatening situation. Its study focuses on affect and emotional recovery (e.g., feeling calm and interested) and is believed to be an immediate and unconscious response to the environment [37]. Attention Restoration Theory (ART) focuses on cognitive responses, proposing that nature assists recovery from attentional fatigue after extended engagement with challenging tasks [38]. The two approaches are viewed as complementary and have areas of overlap, e.g., mental fatigue can be caused by stress. A combination of the two approaches is needed to fully understand the restorative process. Both theories are based on the concept of biophilia [39] which proposes that humans prefer features in the environment that were once favourable for survival and now deliver positive cognitive and affective benefits [33]. As a result, the “perceived naturalness” of a space can be a significant factor, with natural vistas often being rated as more restorative. Conversely “wildness” can be perceived as negative, with fear affecting the restorative properties of the environment [40]. Given the probable increase in technostress during prolonged periods of remote working, it is important to understand whether outdoor natural spaces provide a restorative effect, inhibiting technostress, and how any restorativeness impacts upon work engagement and work–life balance satisfaction. As natural spaces are known to have restorative properties [41], it is often assumed that work engagement will improve following exposure to such spaces. However, Bellini et al. [42] suggested that there is limited research on this assumption; therefore, our study will contribute by providing evidence on the existence of a positive relationship between perceived restorativeness and work engagement.

As far as work–life balance is concerned, researchers argue that exposure to nature and its restorative properties will stimulate a broad set of psychological states, such as positive mood, improved cognitive functioning and higher self-esteem, in short, contributing to increased general life satisfaction [43]. In the field of occupational psychology, work–life balance satisfaction is usually considered as one of the main dimensions contributing to the subjective personal experience of employees’ life satisfaction. Even if work–life balance satisfaction is not a variable traditionally included in existing studies on the psychological restorativeness of nature, we suggest that, beyond triggering psychological states supporting a positive job experience (such as positive work engagement), a higher exposure to natural environments and their restorative properties will also stimulate higher work–life satisfaction. In general, regular breaks from work make detaching from work-related pressures and demands possible, protecting employees from the interference between professional and private life. The element of work–life balance is particularly salient in the context of an increased deployment of remote work arrangements mediated by ICT technology. In the context of the present study, we posit that the possibility to regularly access to restorative natural environments should support personal detachment from the ongoing pressures and intrusion associated with the constant usage of ICT associated with remote-working arrangements.

In line with these arguments, we advance a second set of research hypotheses concerning the positive effects of personal exposure to restorative outdoor natural environments.

**Hypothesis** **2.** *Perceived restorativeness of the outdoor natural environment is a positive predictor of work engagement (h2a) and work–life balance satisfaction (h2b) after controlling for the influence of technostress.*

**Figure 1:**
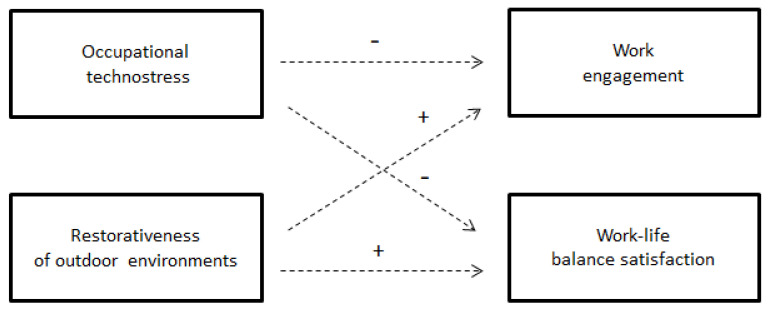
Research model: impact of occupational technostress and perceived restorativeness of natural outdoor environments in conditions of prolonged remote-working arrangements. The symbol “+” entail an hypothesized positive influence of the antecedent to the criteria variable. Conversely, the symbol “−“ entails an hypothesized negative influence of the antecedent.

Figure 1 reports our research hypotheses for the two criteria variables: work engagement and work–life balance satisfaction. Research methods and statistical results are described in the next sections of the article.

## 2. Materials and Methods

### 2.1. Participants and Procedure

Recruitment was carried out using opportunity sampling, via social media platforms (i.e., Twitter, LinkedIn and Facebook) between May and June 2021. The three inclusion criteria were: (a) having exclusively worked remotely from home, through the utilization of information technology, during the period of March 2020 to June 2021, a period characterized by social restrictions in the UK due to the COVID-19 pandemic; (b) having had access to natural spaces whilst working from home during the same period of time; (c) being ordinarily resident in the UK, to ensure that individuals’ experience of lockdown was broadly consistent.

The final sample comprised 109 employed people. The majority (35%) of respondents were aged 36–45 years and 69% self-identified as female; 85% were in full-time employment. Further, 41% of participants reported no working-from-home experience prior to the introduction of social restrictions during the prolonged lockdown in place as a result of the COVID-19 pandemic. Moreover, 63% of respondents reported accessing outdoor space for leisure more than before lockdown, with the majority of visits (81%) conducted on foot and within one mile of home (76%). Additional demographic and occupational information concerning the present research sample is summarized in Table 1. More details about the typology of the natural environment accessed by the research participants are reported in Table 2. The table reports the percentage of research participants who reported having regularly accessed a specific typology of environment during the period of reference in the present study.

### 2.2. Measures

*Perceived Technostress.* Three items from Ragu-Nathan et al.’s [14] technostress questionnaire addressed aspects related to the usage of information technology during the past month of remote working from home, such as ‘cognitive overload induced by the information technology’, ‘technology invasion in one’s own private life’ and ‘perceived complexity of the usage of the information technology’, to work from home. All items were measured on a 5-point Likert scale from 1 (strongly disagree) to 5 (strongly agree). Examples of items *included “I feel like my personal life is being invaded by this technology”* and *“I am forced to change my work habits to adapt to new technologies”*.

*Perceived Restorativeness of Outdoor Natural Spaces (PRN).* This is a modified version of the Perceived Restorativeness Scale [44,45], assessing individual perception of five items associated with restorative qualities assumed to be present in the natural environment. Participants were asked to refer to what extent the natural environment they had mostly accessed during the past month presented a set of characteristics that are conceptualized in the literature as determinants of the subjective experience of psychological restorativeness induced by natural environments. These characteristics included aspects, such as: fascination, being away, coherence, scope and compatibility. Responses from the participants were provided on an 8-point Likert scale from 0 (completely disagree) to 7 (completely agree). Examples of items from this scale were *“places like this are fascinating”* and *“I like to be in places like this to stop thinking about the things I must get done”*.

*Work engagement.* Work engagement was measured using a shortened 3-item scale of the Utrecht Work Engagement Scale [46]. Examples of items include *“at my work I feel bursting with energy”* and *“I am immersed in my work”*. Items were scored on a 7-point frequency scale, rated from 1 (never) to 7 (always).

*Work–Life Balance Satisfaction (WLBS).* One item was adapted from a scale by Fisher et al. [47], scored on a 5-point scale from strongly disagree (1) to strongly agree (5), in order to assess current individual satisfaction between work and life balance elements. An example item included in the scale was: *“I am satisfied with the balance between my job and my personal life right now.”*

*Control Variables.* Participants’ sex (dichotomized, 0 = male and 1 = female) was included in the analysis in light of well-known sex differences in perceptions of work–life balance [48,49]. Similarly, extant literature has found age effects in perceptions of work engagement [50]. Last, exposure to the natural environment was measured with a single item created ad hoc for the present study and was included to reflect varying degrees of contact with natural environments [33]. The participants were asked to provide an estimation of the average of hours spent in a natural environment in the course of a typical week, considering the previous twelve months of lockdown.

## 3. Results

Data were analysed with *Mplus*(Muthén & Muthén, Los Angeles, Ca, USA)*( 8.8* [51] using maximum-likelihood estimation. Preliminary tests suggested that univariate normality assumptions were not violated (skewness range = −0.192–1.87; kurtosis range = −1.45–5.58) and, in accordance with Mahalanobis test, no multivariate outliers were found. A confirmatory factor analysis showed that the measurement model fit the data well (χ^2^(49) = 91.72, CFI = 0.91, RMSEA = 0.08, SRMR = 0.08). As expected (see Table 3), work engagement and perceived restorativeness of outdoors spaces were positively correlated (r = 0.29, *p* < 0.001); WLBS and technostress were also negatively correlated (r = −0.30, *p* < 0.001).

Our main hypotheses were tested using a structural equation model (see Figure 1). As can be seen in Table 4, Hypothesis 1 was supported: technostress was associated with lower work–life balance (β = −0.43, *p* = 0.01), supporting Hypothesis 1b; it was associated with lower work engagement (β = −0.31, *p* = 0.01). Hypothesis 2 was partially supported: psychological restorativeness was positively associated with work engagement (β = 0.19, *p* = 0.02), supporting Hypothesis 2a; however, it was not associated with work–life balance (β = 0.10, *p* = 0.32). Upon reviewer request, we investigated whether the effect of psychological restorativeness was contingent on the levels of exposure to natural environments (i.e., a latent interaction model). However, parameter estimates for the latent interaction term were non-significant (β = −0.03, *p* = 0.69 and β = −0.07, *p* = 0.12 for the work engagement and work–life balance outcomes, respectively).

## 4. Discussion

The purpose of this research was to identify whether access to natural restorative spaces could help to increase occupational wellbeing, in a time characterized by intensive remote working, due to public-health measures in place during the COVID-19 pandemic in the UK. The effect of technostress on the subjective experience of work–life balance satisfaction (WLBS) and work engagement was examined and perceived restorativeness of the natural environment was assessed to identify any beneficial influence on work engagement and WLBS.

The existing literature presents a significant body of evidence that exposure to nature and the outdoors is important for human health and wellbeing [52] and that employees with greater access to natural spaces are more engaged at work [53]. The findings of this study provide further evidence that the perceived restorativeness of outdoor spaces predicted higher levels of work engagement. This is consistent with the Attention Restoration Theory (ART), which posits that indirect attention and “soft” fascination [54] experienced in restorative environments enable higher cognitive functions, such as those required for working, to be replenished after a period of directed attentional fatigue [55]. The results of this study suggest that visits to outdoor natural spaces are psychologically restorative and, thus, heightened work engagement. From an environmental psychology perspective, one interesting question that may arise from these results and which may merit further investigation is to determine if local natural spaces, which have been accessed during a prolonged period of remote working, can be more familiar and, therefore, “softer”, in terms of demands on attention than visiting new spaces, where attention may be more directly focused on not getting lost or being afraid of the “wilderness” [40]. It is possible that familiarity with local natural spaces during a prolonged period of remote working may have provided a positive level of restorativeness that was optimal for recharging attentional energy for work engagement. This also links to the principles of Stress Recovery Theory (SRT), which states that stress recovery occurs as a result of rapid positive affective responses to specific environmental features [26]. As posited by SRT and ART, the environment must have the right balance of interesting features, without being too bland or too overwhelming [56]. Local natural spaces, such as parks and beaches, are likely to provide a good structural diversity of environmental features, without having too many unfamiliar features that would draw interest and require more extensive cognitive processing (reducing the stress-reduction effects).

Conversely, there was no significant relationship between perceived restorativeness of natural spaces and work–life balance satisfaction, as initially hypothesized. This may be due to a number of factors, for example, the presence of non-work-related stressors, such as home schooling, caring responsibilities, inappropriate office setups, loneliness and general uncertainty, of living through a pandemic. As this study did not examine these variables, it is impossible to say with any certainty what effect outdoor restorativeness may have on wider life satisfaction. One factor that is often discussed when considering the health and wellbeing benefits of natural spaces is “dosage”, i.e., frequency, duration and repetition of visits [57]. As perceived restorativeness did not predict work–life balance satisfaction, some consideration should be given to the possibility that, in the context of prolonged remote-work arrangements, there must be a “minimum” dosage of nature exposure to guarantee that its psychological restorative properties can contribute to increased individual personal satisfaction with the interface between their occupational life and the private sphere of their existence. However, it needs to be noted that our statistical analysis took into account the potential influence of the level of self-reported personal exposure to natural environments on the two criteria variables investigated in our research (work engagement; work–life balance satisfaction). In our study, this control variable of ‘personal exposure’ was operationalized in terms of weekly hours of exposure to the natural environment, but without resulting in a statistically significant effect on the criteria variables.

As hypothesised, technostress negatively predicted work–life balance satisfaction, suggesting that increased use of technology in the context of prolonged remote-work arrangements may result in a state of balance dissatisfaction, most likely caused by the perception of interference between the occupational experience with the domain of private life of the employees. This is in line with Ragu-Nathan et al. [14] who also found that technostress decreases job satisfaction. One argument for this effect is that availability of technology at home creates a “work-home” conflict, with remote-work arrangements blurring the boundaries between work and home life [58]. The existing body of literature on the phenomenon of technostress also suggests that ICT can be viewed as “invasive” and lead people to feel pressure to work longer hours [59] and be constantly available [60], negatively impacting on employee satisfaction. Such effects would explain some of the dissatisfaction experienced by participants in the study. A further component of technostress is that of pressure to learn to use new and changing technology [15]. Although many employers introduced virtual communication systems such as Microsoft Teams before the introduction of mandatory remote working, many participants stated in their response in this study that they were using ICT more than previously, a finding supported by Nimrod [20]. Initially, therefore, it is likely that increased use of ICT, and so exposure to technostressors, impacted work–life balance satisfaction. As the working-from-home arrangements progressed with the persistency of the COVID-19 pandemic in the UK, experience of using ICT to work remotely would have increased; therefore, some facets of technostress exposure may have reduced over time. Oksanen et al. [61] supported this, finding that increased use of social media during the pandemic was a predictor of increased technostress, but individuals already used to the platforms were less affected. Therefore, it could be suggested that increasing experience with technology may moderate some factors that lead to technostress. This could account for the finding that technostress accounted for only 8% of the variance in work–life balance satisfaction, suggesting other factors might be involved in shaping employees’ perception of an acceptable balance. It is worth noting that age and sex were not significant predictors of work engagement or work–life balance satisfaction, whereas it has previously been suggested that older workers may be more vulnerable to technostress due to unfamiliarity with the technology [62]. It could be further posited that full-time remote-working arrangements actually force individuals to create a separation between home and work life, particularly if supported by their organizations to do so. Combined with the beneficial effects associated with exposure to restorative natural environments, this could lead to better work engagement during normal working hours and reduce the pressure to be “switched-on” out of these times. This could have implications for post-pandemic organizational and work redesign.

Interestingly, technostress did not provide statistically significant effects in the explanation of the level of employees’ work engagement. This unverified hypothesis could be related to sample-size issues. However, one possible explanation for this is that study participants have adjusted well to the introduction of virtual working, perhaps due to the necessity of having to utilise it on a day-to-day basis to retain contact with colleagues and customers. Bevan et al. [63] found that around one-third of people felt that working-from-home arrangements were motivational, suggesting that the benefits of home working may outweigh a potential negative influence of technostress on work engagement. Benefits cited included no stressful commute, increased autonomy and being in an environment that enhances productivity [62,64]. The findings may also provide initial support for Reese et al.’s [65] suggestion that there may have been a change in place attachment over the course of the pandemic. Place attachment is a construct which is typified by a bond between a person and their “meaningful environments”, including social ties [66]. Reese et al. [65] posited that prolonged remote-working arrangements due to the persistent pandemic lockdown resulted in a sudden change in place attachment and sense of self-identity. In this scenario, the use of ICT whilst working from home may have enabled new place attachments to form, including the home as a workplace, a greater connection to one’s local community spaces and development of new social groups, at home and at work, through use of online platforms. The effect may even have been heightened by the shared collective coping experience with the restrictions induced by the persistency of the COVID-19 pandemic. Such bonds could explain why technostress did not negatively impact work engagement, and why reports are suggesting that growing numbers of people now prefer working-from-home arrangements [67]. It is, therefore, possible that work location attachment, as well as the other benefits brought by the flexibility of remote-work arrangements, may be overriding the negative effects of technostress. Combined with the finding that technostress did cause a small but significant decrease in work–life balance satisfaction, it is important that future “hybrid models” of working feature time to access natural spaces built into the working day.

Study limitations. This study aimed to contribute to understanding how the influence of psychologically restorative properties in natural environments support positive psychological states (such as work engagement) in prolonged occupational experiences of remote-working arrangements. However, in addition to the contributions to our understanding of the effects of prolonged remote-working arrangements, like all the empirical studies, this research presents a few limitations. In this subsection, these aspects of the study will be briefly reviewed.

First, our design was cross-sectional. Therefore, causal interpretation of the results from the structural equation models cannot be drawn from the data. Further, reverse causal effects cannot be excluded (i.e., positive work engagement leading to increased sensitivity to psychological restorativeness of natural spaces, or a positive work–life balance leading to a decreased vulnerability to technostress). Beyond the prudence in the interpretation of the results emerging from our analyses, longitudinal studies could also aid in the understanding of whether the observed restorative effects of nature are maintained over time.

Second, only a relatively low proportion of the variance for both technostress and perceived restorativeness is explained by the statistical analyses. Therefore, it is likely that there are other important factors in predicting work engagement and work–life balance satisfaction. The previous discussion has highlighted some topics that might be of significance (such as other sources of occupational stress or the restorative properties of the home/work environment), and is supported by a growing body of literature, which identifies multiple stressors that can all impact work engagement [68]. These, and potentially other variables (e.g., mental and physical wellbeing), are worthy of future scrutiny.

Third, we need to recognise that not all “natural spaces” may have similar benefits, and the spaces experienced by our research participants may not represent all the “natural spaces” on an ideal continuum of restorativeness benefits. Even if our research participants reported various types of natural spaces they regularly accessed during the twelve-month lockdown period, no specific analyses were conducted on the present sample to investigate the differential effect of distinct kinds of natural environments on the subjective experience of psychological restorativeness. Future replications of the present study could be designed to address this research gap.

Future research avenues. The results provided some interesting insights into the effects of technostress and perceived restorativeness of natural spaces on work engagement and work–life balance satisfaction. This study was largely grounded in the environmental psychological theories of attention and stress reduction. A first future research avenue should focus on the role of the psychology of individual differences in response not only to the natural environment (and its perceived restorativeness), but also in relation to personal preferences toward specific forms of work (i.e., shift work), or in relation to personal attitudes and dispositions to work as part of virtual teams, or, in contrast, in relation to personal inclinations to lonely work settings. All of these aspects are worthy of further scrutiny, particularly given the growing evidence base surrounding the importance of these factors during the prolonged remote-work arrangements during the recent lockdowns in response to the global COVID-19 pandemic. Such investigations could draw from a wide body of theory, including personality, gender studies, inequality and organizational psychology. The research could be further expanded to include data on general wellbeing, linking to the wider evidence base on the effects of nature on mental and physical health. Second, it would also be interesting to repeat this study now that the prolonged remote-working arrangements due to the recent lockdown policies related to the COVID-19 pandemic have ended, in order to assess whether any lasting changes can be observed, for example, how persistent and enduring hybrid/home-working models will become, and whether any sustained increase in remote/home working will result in individuals increasing their exposure to natural spaces in their daily work practices. A longitudinal assessment of any changes in technostress levels as people adapt to remote working would also be of interest. There is also the opportunity to explore, in more depth, whether the attachment to place, and so identity, has permanently shifted, and if this is beneficial for work productivity, social cohesion and inspiring more pro-environmental behaviours, as people feel more connection to their local area. Third, beyond the specific focus of our study on the restorative properties of outdoor natural environments, the restorativeness of indoor spaces, particularly the home, office and home office, also requires further scrutiny, especially for those for whom prolonged remote-working arrangements have been a negative experience. This is significant for organizations who are likely to move to a blended model of remote- and office-based working post-pandemic. Fourth, at the time of data collection, this sample’s remote-work arrangements had been ongoing for around 16 months. It would be interesting to examine whether people’s relationships with their local spaces changed over time and whether there is now more appetite for exploration of wilder areas, to actively gain the stimulation of visiting new places and a more intensive nature experience. Finally, due to the research focus of our study on the psychological impacts of outdoor restorativeness on two occupational-related psychological constructs (i.e., work engagement and work–life balance satisfaction), our research found its conceptual foundations mainly in the literature related to occupational psychology and environmental psychology. However, future studies could attempt to integrate these research fields with other disciplines that have also contributed to the scientific understanding of the effects of nature and outdoor spaces on wellbeing, for example, the research fields of leisure and recreation studies [69,70].

## 5. Conclusions

The findings suggest that access to outdoor natural spaces can help support positive work engagement during prolonged periods of home working characterized by an increased usage of ICT, which may lead to a rise in technostress. The results revealed that technostress negatively impacts work–life balance satisfaction, whilst exposure to natural spaces stimulated positive work engagement. These outcomes contribute to the rapidly expanding literature regarding the use of ICT in remote working during the COVID-19 pandemic, as well as providing further support for nature being a restorative factor, which can contribute to the maintenance of positive psychological states, such as work engagement, in the presence of remote-work arrangements. The results pave the way for further exploration of the changes that have taken place in people’s working lives as a result of the pandemic and how new ways of working in a post-pandemic society can incorporate and develop people’s access to restorative natural spaces for their own productivity and wellbeing.

## Figures and Tables

**Table 1:** Research sample: demographic and occupational characteristics.

Sex	Female: 69.2%	Male: 29.4%	Other: 2.6%	Not said: 0.8%
Age	36–45 years: 35%	26–35 years: 27%	46–55 years: 23%	More than 55: 12.7%
Typology of employment	Office administration: 52.1%	Education sector: 19.8%	Healthcare sector: 12.4%	Other 15.7%
Employment status	Full-time employment: 84.3%	Part-time employment: 12.4%	Self-employment:3.3%	

**Table 2:** Typology of natural environment regularly visited by the participants.

Typology of Environment	Percentage
Parkland (e.g., grass, meadow, heath, widely spaced trees)	54%
Woodland (e.g., native species, coniferous plantation)	47%
Water (e.g., river, stream/burn, pond, canal, etc.)	45%
Field and farmland	25%
Recreation facilities (e.g., sports facilities, amenity grasslands, children’s play areas)	25%
Beach (any type)/Coastal (sea cliffs)	13%
Hills, mountains, wild lands	12%
Wetland (marsh, bog, etc.)	9%

*Note*. The percentage indices refer to the participants who report regularly visiting each of the listed typologies of natural environments

**Table 3:** Descriptive and correlation statistics.

	M	SD	1	2	3	4	5	6	7
1. Psych. Restorativeness	5.92	1.29	*(0.92)*						
2. Technostress	2.54	0.82	0.02	*(0.87)*					
3. Work Engagement	4.78	1.04	0.29 ***	−0.14	*(0.93)*				
4. Work–life Balance	3.41	1.00	0.10	−0.3 ***	0.59 ***				
5. Age Group	3.21	1.15	−0.17	−0.12	−0.08	0.11			
6. Sex	0.69	0.46	0.11	0.07	0.05	0.02	0.05		
7. Exposure to Nature	3.51	1.38	0.48 ***	−0.09	0.20	0.09	0.06	0.05	

*Note. N* = 118 *** *p* < 0.01; Cronbach’s Alphas indices are reported in italics and in parentheses in the diagonal of the table.

**Table 4:** Main results: hypothesis test.

Predictors	Work Engagement			Work–Life Balance		
	Estimate	SE	*p*-Value	Estimate	SE	*p*-Value
Psychological Restorativeness	0.19	0.08	0.01	0.10	0.10	0.31
Technostress	−0.31	0.13	0.01	−0.43	0.17	0.01
Age	−0.08	0.07	0.27	0.06	0.11	0.59
Sex	0.24	0.18	0.18	0.18	0.26	0.49
Exposure to Natural Environments	0.04	0.06	0.50	0.00	0.09	0.96
R^2^	0.23			0.23		

*Note.* Parameter estimates are unstandardized.

## Data Availability

The data presented in this study are available on request from the corresponding author.
